# *H*-type Ce_2_[Si_2_O_7_]

**DOI:** 10.1107/S2414314623005916

**Published:** 2023-07-07

**Authors:** Ralf Jules Christian Locke, Maria Weis, Thomas Schleid

**Affiliations:** a Institut für Anorganische Chemie, Universität Stuttgart, Pfaffenwaldring 55, 70569 Stuttgart, Germany; Vienna University of Technology, Austria

**Keywords:** crystal structure, oxidodisilicate, rare-earth metal, cerium, isotypism

## Abstract

The title compound, dicerium(III) oxidodisilicate, Ce_2_[Si_2_O_7_], was obtained as a by-product in its *H*-type structure after attempts to synthesize CeSb_2_O_4_Cl from fused silica ampoules. It crystallizes isotypically with *H*-La_2_[Si_2_O_7_]. The four crystallographically distinct Ce^III^ cations form distorted square anti­prisms, capped square anti­prisms, and bicapped square anti­prisms as coordination polyhedra consisting of oxygen atoms. Four crystallographically different silicon atoms recruit the centers of two different isolated [Si_2_O_7_]^6–^ units.

## Abstract 2

Ce_2_[Si_2_O_7_] was obtained in its *H*-type structure and crystallizes isotypically with *H*-La_2_[Si_2_O_7_].

## Graphical abstract

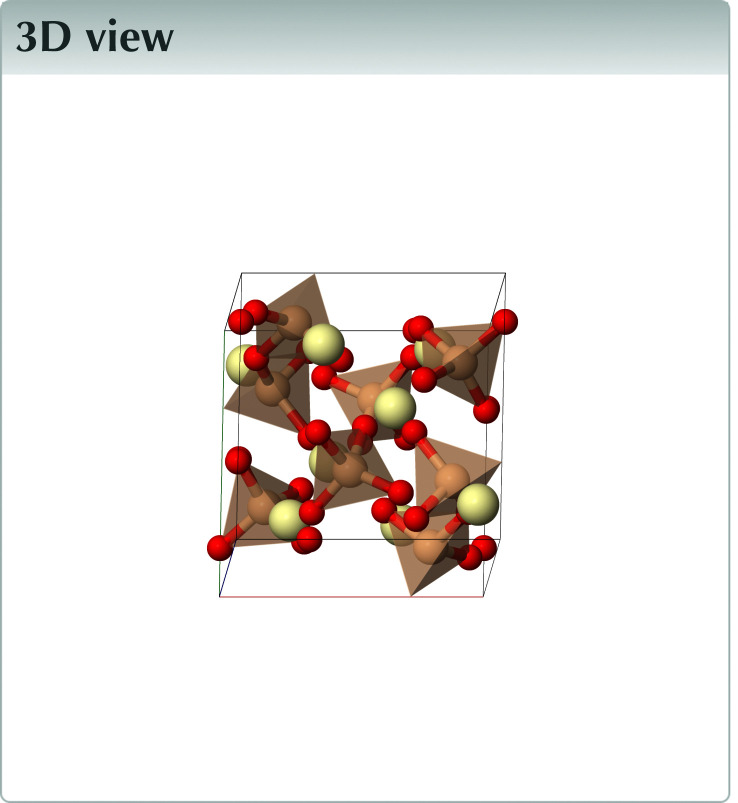

## Structure description

**Figure 1:**
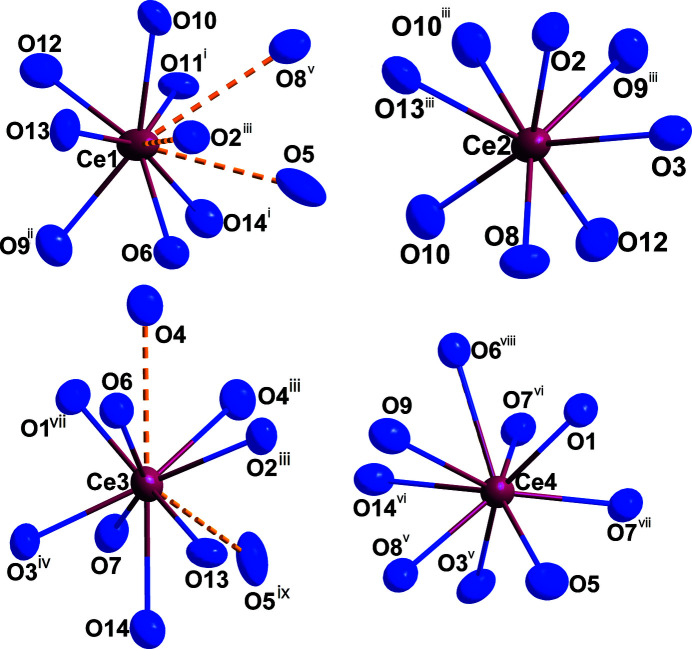
Oxygen environment of the four crystallographically different Ce^III^ cations in *H*-type Ce_2_[Si_2_O_7_]. The yellow dotted bonds reflect cerium–oxygen distances longer than 3.0 Å. Displacement ellipsoids are drawn at the 95% probability level. Symmetry codes refer to Table 1 ▸.

**Figure 2:**
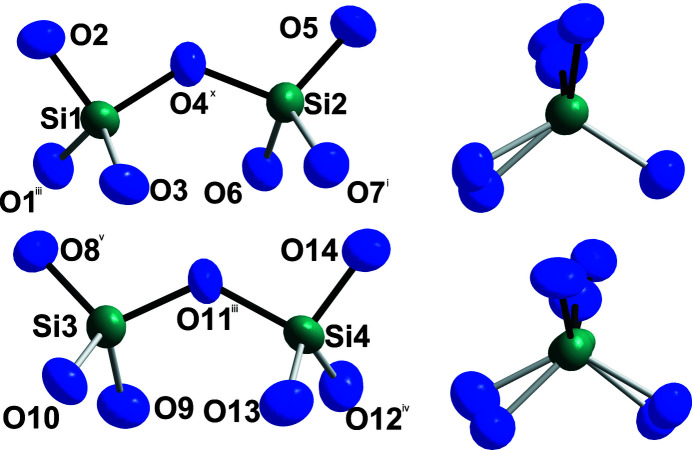
The two distinct oxidodisilicate anions [Si_2_O_7_]^6–^ made of two vertex-connected [SiO_4_]^4–^ tetra­hedra in *H*-type Ce_2_[Si_2_O_7_], where the position of the oxygen atoms define a backbone arrangement (*left*), and their *Newman* projection (*right*). Displacement ellipsoids are drawn at the 95% probability level. Symmetry codes refer to Table 1 ▸.

**Figure 3:**
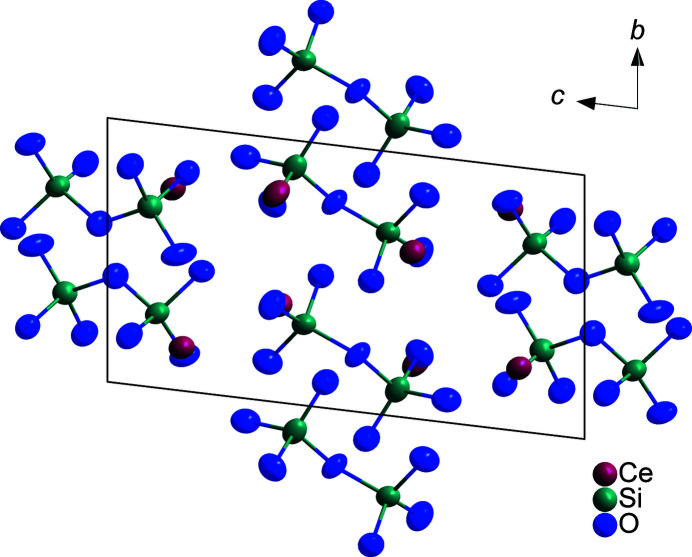
View of the triclinic crystal structure of *H*-type Ce_2_[Si_2_O_7_] along [100] emphasizing the discrete [Si_2_O_7_]^6–^ anions. Displacement ellipsoids are drawn at the 95% probability level.

*H*-type Ce_2_[Si_2_O_7_], like *H*-La_2_[Si_2_O_7_] (Müller-Bunz & Schleid, 2000 ▸), crystallizes isotypically with the triclinic form of potassium dichromate [K_2_[Cr_2_O_7_]; Brandon & Brown, 1968 ▸] in the space group *P* 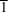 . According to the single-crystal X-ray structure analysis, four crystallographically distinct Ce^III^ cations with coordination numbers ranging from eight to ten are present (Fig. 1 ▸), with oxygen atoms forming distorted square anti­prisms (Ce2), capped square anti­prisms (Ce4), and bicapped square anti­prisms (Ce1 and Ce3) as coordination polyhedra. The cerium–oxygen distances *d*(Ce—O) cover an inter­val from 2.366 (4) to 2.817 (4) Å (Table 1 ▸) *plus* 3.11 (4)–3.34 (4) Å to most caps. All oxygen atoms belong to pyroanionic oxidodisilicate anions [Si_2_O_7_]^6–^ (Fig. 2 ▸), each consisting of two vertex-connected [SiO_4_]^4–^ tetra­hedra. Here, four crystallographically different silicon atoms recruit the centers of these two isolated [Si_2_O_7_]^6–^ units [*d*(Si—O) = 1.588 (4)–1.676 (4) Å (Table 1 ▸); ∠(O—Si—O) = 100.67 (19)–117.4 (2)°]. Both exhibit an ecliptical conformation with Si—O—Si angles of 129.2 (2) and 128.8 (2)°, leading to a backbone-to-backbone alignment of the Si—O—Si bridges. The silicon–oxygen distances are in the usual range for this element combination, with slightly longer contacts to the bridging oxygen atoms (Table 1 ▸). The shortest, of course non-bonding, cerium–silicon distances of 3.2118 (14)–3.3391 (14) Å reflect the close proximity of Ce^III^ to the discrete [Si_2_O_7_]^6–^ anions. Figure 3 ▸ shows the content of an extended unit-cell with highlighted [Si_2_O_7_]^6–^ bi­tetra­hedra. The similarity to the other so-far known polymorphs of Ce_2_[Si_2_O_7_] [*A*- (Kępiński *et al.*, 2002 ▸; Deng & Ibers, 2005 ▸) and *G*-type (Tas & Akinc, 1994 ▸; Christensen, 1994 ▸; Christensen & Hazell, 1994 ▸) and even *I*-type Ce_2_Si_2_O_7_ (≡ Ce_6_[Si_4_O_13_][SiO_4_]_2_) (Kępiński *et al.*, 2002 ▸)] is striking and will be discussed in an upcoming review article (Hartenbach *et al.*, 2023 ▸) as a follow up of the pioneering one by Felsche (1970 ▸).

## Synthesis and crystallization

Single crystals of *H*-Ce_2_[Si_2_O_7_] were obtained as a by-product during the synthesis of CeSb_2_O_4_Cl (Locke, 2023 ▸; Weis, 2023 ▸) by reacting Ce_2_O_3_ with fused silica (SiO_2_) as reaction vessel at a temperature of 1023 K, taking advantage of the presumed mineralizers Sb_2_O_3_ and CeCl_3_. The transparent, colorless crystals exhibit a platelet-like habit.

## Refinement

Crystal data, data collection and structure refinement details are summarized in Table 2 ▸.

## Supplementary Material

Crystal structure: contains datablock(s) I, 1R. DOI: 10.1107/S2414314623005916/wm4192sup1.cif

Structure factors: contains datablock(s) I. DOI: 10.1107/S2414314623005916/wm4192Isup2.hkl

CCDC reference: 2222660

Additional supporting information:  crystallographic information; 3D view; checkCIF report

## Figures and Tables

**Table 1:** Selected bond lengths (Å)

Ce1—O6	2.386 (4)	Ce3—O1^vii^	2.396 (4)
Ce1—O13	2.439 (4)	Ce3—O7	2.457 (4)
Ce1—O12	2.445 (4)	Ce3—O2^iii^	2.490 (4)
Ce1—O10	2.480 (4)	Ce3—O3^iv^	2.534 (4)
Ce1—O14^i^	2.486 (4)	Ce3—O6	2.555 (4)
Ce1—O9^ii^	2.516 (4)	Ce3—O13	2.632 (4)
Ce1—O11^i^	2.663 (4)	Ce3—O14	2.687 (4)
Ce1—Si4^i^	3.2597 (15)	Ce3—O4^iii^	2.705 (4)
Ce1—Si2	3.3340 (15)	Ce3—Si4	3.2767 (15)
Ce1—Si3	3.4775 (14)	Ce3—Si1^iii^	3.3138 (14)
Ce1—Ce3	3.9086 (4)	Ce3—Si2^ix^	3.3545 (14)
Ce1—Ce4^ii^	3.9449 (4)	Ce3—Si1^x^	3.4591 (15)
Ce2—O8	2.366 (4)	Si1—O1^iii^	1.592 (4)
Ce2—O2	2.370 (4)	Si1—O2	1.624 (4)
Ce2—O12	2.376 (4)	Si1—O3	1.632 (4)
Ce2—O10	2.494 (4)	Si1—O4	1.664 (4)
Ce2—O10^iii^	2.526 (4)	Si1—Ce3^iii^	3.3138 (14)
Ce2—O13^iii^	2.643 (4)	Si1—Ce4^iii^	3.4549 (14)
Ce2—O9^iii^	2.675 (4)	Si1—Ce3^xi^	3.4591 (15)
Ce2—O3	2.817 (4)	Si2—O5	1.589 (4)
Ce2—Si1	3.2118 (14)	Si2—O7^i^	1.636 (4)
Ce2—Si3^iii^	3.2386 (15)	Si2—O6	1.642 (4)
Ce2—Si4^iv^	3.4514 (15)	Si2—O4^x^	1.660 (4)
Ce2—Ce1^iii^	3.9450 (4)	Si2—Ce4^ii^	3.3391 (14)
Ce4—O5	2.415 (4)	Si2—Ce3^i^	3.3544 (14)
Ce4—O1	2.420 (4)	Si3—O8^v^	1.595 (4)
Ce4—O3^v^	2.517 (4)	Si3—O9	1.632 (4)
Ce4—O7^vi^	2.576 (4)	Si3—O10	1.641 (4)
Ce4—O7^vii^	2.603 (4)	Si3—O11^iii^	1.648 (4)
Ce4—O8^v^	2.655 (4)	Si3—Ce2^iii^	3.2386 (15)
Ce4—O14^vi^	2.681 (4)	Si4—O12^iv^	1.588 (4)
Ce4—O9	2.749 (4)	Si4—O13	1.620 (4)
Ce4—O6^viii^	2.812 (4)	Si4—O14	1.631 (4)
Ce4—Si3	3.2807 (14)	Si4—O11	1.676 (4)
Ce4—Si2^viii^	3.3391 (14)	Si4—Ce1^ix^	3.2597 (15)
Ce4—Si1^iii^	3.4549 (14)	Si4—Ce2^iv^	3.4514 (15)

Symmetry codes: (i) 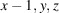 ; (ii) 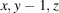 ; (iii) 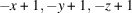 ; (iv) 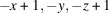 ; (v) 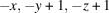 ; (vi) 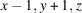 ; (vii) 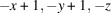 ; (viii) 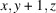 ; (ix) 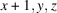 ; (x) 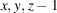 ; (xi) 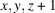 .

**Table 2:** Experimental details

Crystal data	
Chemical formula	Ce_2_[Si_2_O_7_]
*M*_r_	448.42
Crystal system, space group	Triclinic, *P* 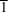
Temperature (K)	293
*a*, *b*, *c* (Å)	6.7671 (4), 6.8228 (4), 12.4237 (8)
α, β, γ (°)	83.116 (2), 87.975 (2), 88.854 (2)
*V* (Å^3^)	569.05 (6)
*Z*	4
Radiation type	Mo *K*α
μ (mm^−1^)	16.20
Crystal size (mm)	0.05 × 0.03 × 0.01

Data collection	
Diffractometer	Stadi-Vari
Absorption correction	Numerical (*LANA*; Koziskova *et al.*, 2016 ▸)
*T*_min_, *T*_max_	0.414, 0.808
No. of measured, independent and observed [*I* > 2σ(*I*)] reflections	23791, 4046, 3376
*R*_int_	0.035
(sin θ/λ)_max_ (Å^−1^)	0.767

Refinement	
*R*[*F* ^2^ > 2σ(*F* ^2^)], *wR*(*F* ^2^), *S*	0.030, 0.075, 1.00
No. of reflections	4046
No. of parameters	199
Δρ_max_, Δρ_min_ (e Å^−3^)	2.54, −2.81

Computer programs: *X-AREA* (Stoe, 2020 ▸), *SHELXS97* and *SHELXL97* (Sheldrick, 2008 ▸) and *DIAMOND* (Brandenburg & Putz, 2005 ▸).

## full crystallographic data

### Crystal data

**Table:**

Ce_2_[Si_2_O_7_]	*Z* = 4
*M**_r_* = 448.42	*F*(000) = 800
Triclinic, *P*1	*D*_x_ = 5.234 Mg m^−^^3^
Hall symbol: -P 1	Mo *K*α radiation, λ = 0.71073 Å
*a* = 6.7671 (4) Å	Cell parameters from 23784 reflections
*b* = 6.8228 (4) Å	θ = 1.7–33.1°
*c* = 12.4237 (8) Å	µ = 16.20 mm^−^^1^
α = 83.116 (2)°	*T* = 293 K
β = 87.975 (2)°	Platelet, colourless
γ = 88.854 (2)°	0.05 × 0.03 × 0.01 mm
*V* = 569.05 (6) Å^3^	

### Data collection

**Table:**

Stadi-Vari diffractometer	4046 independent reflections
Radiation source: fine-focus sealed tube	3376 reflections with *I* > 2σ(*I*)
Graphite monochromator	*R*_int_ = 0.035
Detector resolution: 5.81 pixels mm^-1^	θ_max_ = 33.0°, θ_min_ = 1.7°
DECTRIS PILATUS 200K scans	*h* = −10→10
Absorption correction: numerical (*LANA*; Koziskova *et al.*, 2016)	*k* = −10→10
*T*_min_ = 0.414, *T*_max_ = 0.808	*l* = −19→19
23791 measured reflections	

### Refinement

**Table:**

Refinement on *F*^2^	0 restraints
Least-squares matrix: full	Primary atom site location: structure-invariant direct methods
*R*[*F*^2^ > 2σ(*F*^2^)] = 0.030	Secondary atom site location: difference Fourier map
*wR*(*F*^2^) = 0.075	*w* = 1/[σ^2^(*F*_o_^2^) + (0.0498*P*)^2^] where *P* = (*F*_o_^2^ + 2*F*_c_^2^)/3
*S* = 1.00	(Δ/σ)_max_ < 0.001
4046 reflections	Δρ_max_ = 2.54 e Å^−^^3^
199 parameters	Δρ_min_ = −2.81 e Å^−^^3^

### Special details

**Table:**

Geometry. All esds (except the esd in the dihedral angle between two l.s. planes) are estimated using the full covariance matrix. The cell esds are taken into account individually in the estimation of esds in distances, angles and torsion angles; correlations between esds in cell parameters are only used when they are defined by crystal symmetry. An approximate (isotropic) treatment of cell esds is used for estimating esds involving l.s. planes.
Refinement. Refinement of F^2^ against ALL reflections. The weighted R-factor wR and goodness of fit S are based on F^2^, conventional R-factors R are based on F, with F set to zero for negative F^2^. The threshold expression of F^2^ > 2sigma(F^2^) is used only for calculating R-factors(gt) etc. and is not relevant to the choice of reflections for refinement. R-factors based on F^2^ are statistically about twice as large as those based on F, and R- factors based on ALL data will be even larger.

### Fractional atomic coordinates and isotropic or equivalent isotropic displacement parameters (Å^2^)

**Table:**

	*x*	*y*	*z*	*U*_iso_*/*U*_eq_	
Ce1	0.23961 (4)	0.21088 (4)	0.35322 (2)	0.01505 (7)	
Ce2	0.37019 (4)	0.36951 (4)	0.63911 (2)	0.01288 (7)	
Ce4	0.07768 (4)	0.83629 (4)	0.15567 (2)	0.01239 (7)	
Ce3	0.67603 (4)	0.23866 (4)	0.13758 (2)	0.01296 (7)	
Si1	0.4321 (2)	0.2825 (2)	0.89563 (11)	0.0115 (2)	
Si2	0.1605 (2)	0.3171 (2)	0.08912 (11)	0.0120 (2)	
Si3	0.1616 (2)	0.6927 (2)	0.41176 (11)	0.0116 (2)	
Si4	0.7737 (2)	0.1022 (2)	0.39232 (11)	0.0122 (2)	
O1	0.3784 (6)	0.8159 (5)	0.0460 (3)	0.0138 (7)	
O2	0.4898 (6)	0.4620 (5)	0.8025 (3)	0.0142 (7)	
O3	0.2938 (6)	0.1348 (5)	0.8380 (3)	0.0152 (7)	
O4	0.3020 (6)	0.4046 (5)	0.9831 (3)	0.0144 (7)	
O5	0.0549 (6)	0.4850 (6)	0.1496 (3)	0.0204 (8)	
O6	0.3068 (6)	0.1720 (5)	0.1671 (3)	0.0135 (7)	
O7	0.9958 (6)	0.1729 (6)	0.0479 (3)	0.0151 (7)	
O8	0.0207 (6)	0.3667 (6)	0.6549 (3)	0.0157 (7)	
O9	0.2996 (6)	0.8508 (5)	0.3356 (3)	0.0161 (7)	
O10	0.3069 (6)	0.5021 (5)	0.4473 (3)	0.0144 (7)	
O11	0.9092 (6)	0.2162 (5)	0.4758 (3)	0.0140 (7)	
O12	0.3041 (6)	0.0981 (6)	0.5432 (3)	0.0163 (7)	
O13	0.5976 (6)	0.2494 (6)	0.3458 (3)	0.0156 (7)	
O14	0.9272 (6)	0.0858 (5)	0.2895 (3)	0.0152 (7)	

### Atomic displacement parameters (Å^2^)

**Table:**

	*U*^11^	*U*^22^	*U*^33^	*U*^12^	*U*^13^	*U*^23^
Ce1	0.01240 (13)	0.01724 (13)	0.01669 (14)	−0.00301 (10)	0.00049 (10)	−0.00672 (10)
Ce2	0.01202 (13)	0.01287 (12)	0.01415 (13)	−0.00143 (9)	−0.00067 (10)	−0.00300 (9)
Ce4	0.01122 (13)	0.01164 (12)	0.01448 (13)	0.00012 (9)	0.00034 (9)	−0.00260 (9)
Ce3	0.01290 (13)	0.01218 (12)	0.01400 (13)	0.00150 (9)	0.00060 (10)	−0.00289 (9)
Si1	0.0113 (6)	0.0107 (6)	0.0129 (6)	−0.0003 (5)	−0.0001 (5)	−0.0034 (4)
Si2	0.0102 (6)	0.0121 (6)	0.0135 (6)	−0.0006 (5)	0.0002 (5)	−0.0015 (5)
Si3	0.0121 (6)	0.0099 (6)	0.0128 (6)	0.0008 (5)	−0.0011 (5)	−0.0018 (5)
Si4	0.0092 (6)	0.0148 (6)	0.0131 (6)	0.0026 (5)	−0.0011 (5)	−0.0041 (5)
O1	0.0108 (16)	0.0161 (16)	0.0153 (17)	−0.0003 (13)	−0.0006 (13)	−0.0048 (13)
O2	0.0168 (18)	0.0111 (15)	0.0150 (16)	−0.0021 (13)	−0.0014 (13)	−0.0019 (12)
O3	0.0141 (17)	0.0124 (16)	0.0202 (18)	−0.0004 (13)	−0.0037 (14)	−0.0058 (13)
O4	0.0132 (17)	0.0150 (16)	0.0147 (17)	−0.0007 (13)	0.0043 (13)	−0.0025 (13)
O5	0.021 (2)	0.0129 (17)	0.028 (2)	−0.0013 (15)	0.0074 (16)	−0.0077 (15)
O6	0.0129 (17)	0.0142 (16)	0.0135 (16)	0.0006 (13)	0.0004 (13)	−0.0032 (13)
O7	0.0149 (17)	0.0154 (16)	0.0158 (17)	−0.0025 (14)	−0.0009 (14)	−0.0044 (13)
O8	0.0119 (17)	0.0204 (18)	0.0159 (17)	−0.0027 (14)	−0.0009 (13)	−0.0058 (14)
O9	0.0142 (18)	0.0142 (17)	0.0195 (18)	0.0007 (13)	0.0016 (14)	−0.0003 (13)
O10	0.0152 (18)	0.0130 (16)	0.0150 (17)	0.0036 (13)	0.0010 (13)	−0.0023 (13)
O11	0.0144 (17)	0.0149 (16)	0.0141 (16)	−0.0007 (13)	0.0008 (13)	−0.0074 (13)
O12	0.0189 (19)	0.0148 (17)	0.0156 (17)	−0.0027 (14)	0.0010 (14)	−0.0032 (13)
O13	0.0131 (17)	0.0162 (17)	0.0169 (17)	0.0012 (13)	−0.0042 (14)	0.0013 (13)
O14	0.0130 (17)	0.0132 (16)	0.0192 (18)	−0.0012 (13)	0.0006 (14)	−0.0018 (13)

### Geometric parameters (Å, º)

**Table:**

Ce1—O6	2.386 (4)	Ce3—Si1^x^	3.4591 (15)
Ce1—O13	2.439 (4)	Si1—O1^iii^	1.592 (4)
Ce1—O12	2.445 (4)	Si1—O2	1.624 (4)
Ce1—O10	2.480 (4)	Si1—O3	1.632 (4)
Ce1—O14^i^	2.486 (4)	Si1—O4	1.664 (4)
Ce1—O9^ii^	2.516 (4)	Si1—Ce3^iii^	3.3138 (14)
Ce1—O11^i^	2.663 (4)	Si1—Ce4^iii^	3.4549 (14)
Ce1—Si4^i^	3.2597 (15)	Si1—Ce3^xi^	3.4591 (15)
Ce1—Si2	3.3340 (15)	Si2—O5	1.589 (4)
Ce1—Si3	3.4775 (14)	Si2—O7^i^	1.636 (4)
Ce1—Ce3	3.9086 (4)	Si2—O6	1.642 (4)
Ce1—Ce4^ii^	3.9449 (4)	Si2—O4^x^	1.660 (4)
Ce2—O8	2.366 (4)	Si2—Ce4^ii^	3.3391 (14)
Ce2—O2	2.370 (4)	Si2—Ce3^i^	3.3544 (14)
Ce2—O12	2.376 (4)	Si3—O8^v^	1.595 (4)
Ce2—O10	2.494 (4)	Si3—O9	1.632 (4)
Ce2—O10^iii^	2.526 (4)	Si3—O10	1.641 (4)
Ce2—O13^iii^	2.643 (4)	Si3—O11^iii^	1.648 (4)
Ce2—O9^iii^	2.675 (4)	Si3—Ce2^iii^	3.2386 (15)
Ce2—O3	2.817 (4)	Si4—O12^iv^	1.588 (4)
Ce2—Si1	3.2118 (14)	Si4—O13	1.620 (4)
Ce2—Si3^iii^	3.2386 (15)	Si4—O14	1.631 (4)
Ce2—Si4^iv^	3.4514 (15)	Si4—O11	1.676 (4)
Ce2—Ce1^iii^	3.9450 (4)	Si4—Ce1^ix^	3.2597 (15)
Ce4—O5	2.415 (4)	Si4—Ce2^iv^	3.4514 (15)
Ce4—O1	2.420 (4)	O1—Si1^iii^	1.593 (4)
Ce4—O3^v^	2.517 (4)	O1—Ce3^vii^	2.396 (4)
Ce4—O7^vi^	2.576 (4)	O2—Ce3^iii^	2.490 (4)
Ce4—O7^vii^	2.603 (4)	O3—Ce4^v^	2.517 (4)
Ce4—O8^v^	2.655 (4)	O3—Ce3^iv^	2.534 (4)
Ce4—O14^vi^	2.681 (4)	O4—Si2^xi^	1.660 (4)
Ce4—O9	2.749 (4)	O4—Ce3^iii^	2.705 (4)
Ce4—O6^viii^	2.812 (4)	O6—Ce4^ii^	2.812 (4)
Ce4—Si3	3.2807 (14)	O7—Si2^ix^	1.636 (4)
Ce4—Si2^viii^	3.3391 (14)	O7—Ce4^xii^	2.576 (4)
Ce4—Si1^iii^	3.4549 (14)	O7—Ce4^vii^	2.603 (4)
Ce3—O1^vii^	2.396 (4)	O8—Si3^v^	1.595 (4)
Ce3—O7	2.457 (4)	O8—Ce4^v^	2.655 (4)
Ce3—O2^iii^	2.490 (4)	O9—Ce1^viii^	2.516 (4)
Ce3—O3^iv^	2.534 (4)	O9—Ce2^iii^	2.675 (4)
Ce3—O6	2.555 (4)	O10—Ce2^iii^	2.526 (4)
Ce3—O13	2.632 (4)	O11—Si3^iii^	1.648 (4)
Ce3—O14	2.687 (4)	O11—Ce1^ix^	2.663 (4)
Ce3—O4^iii^	2.705 (4)	O12—Si4^iv^	1.588 (4)
Ce3—Si4	3.2767 (15)	O13—Ce2^iii^	2.643 (4)
Ce3—Si1^iii^	3.3138 (14)	O14—Ce1^ix^	2.486 (4)
Ce3—Si2^ix^	3.3545 (14)	O14—Ce4^xii^	2.681 (4)
			
O6—Ce1—O13	80.46 (13)	O1^vii^—Ce3—O13	158.15 (12)
O6—Ce1—O12	147.59 (13)	O7—Ce3—O13	128.05 (12)
O13—Ce1—O12	81.45 (13)	O2^iii^—Ce3—O13	61.06 (12)
O6—Ce1—O10	127.63 (12)	O3^iv^—Ce3—O13	92.21 (12)
O13—Ce1—O10	73.40 (13)	O6—Ce3—O13	73.85 (12)
O12—Ce1—O10	71.25 (12)	O1^vii^—Ce3—O14	134.41 (12)
O6—Ce1—O14^i^	75.12 (13)	O7—Ce3—O14	71.46 (12)
O13—Ce1—O14^i^	152.98 (13)	O2^iii^—Ce3—O14	109.22 (12)
O12—Ce1—O14^i^	113.96 (13)	O3^iv^—Ce3—O14	64.76 (12)
O10—Ce1—O14^i^	131.49 (12)	O6—Ce3—O14	119.09 (12)
O6—Ce1—O9^ii^	70.85 (13)	O13—Ce3—O14	58.17 (12)
O13—Ce1—O9^ii^	87.75 (13)	O1^vii^—Ce3—O4^iii^	73.44 (12)
O12—Ce1—O9^ii^	81.79 (12)	O7—Ce3—O4^iii^	84.49 (12)
O10—Ce1—O9^ii^	148.87 (13)	O2^iii^—Ce3—O4^iii^	58.16 (11)
O14^i^—Ce1—O9^ii^	73.55 (13)	O3^iv^—Ce3—O4^iii^	152.59 (11)
O6—Ce1—O11^i^	133.66 (12)	O6—Ce3—O4^iii^	105.35 (12)
O13—Ce1—O11^i^	145.82 (12)	O13—Ce3—O4^iii^	115.11 (11)
O12—Ce1—O11^i^	69.02 (12)	O14—Ce3—O4^iii^	127.02 (11)
O10—Ce1—O11^i^	80.90 (11)	O1^vii^—Ce3—Si4	154.61 (9)
O14^i^—Ce1—O11^i^	59.92 (12)	O7—Ce3—Si4	101.12 (9)
O9^ii^—Ce1—O11^i^	104.08 (12)	O2^iii^—Ce3—Si4	87.58 (9)
O6—Ce1—Si4^i^	104.09 (9)	O3^iv^—Ce3—Si4	72.78 (9)
O13—Ce1—Si4^i^	171.06 (9)	O6—Ce3—Si4	93.50 (9)
O12—Ce1—Si4^i^	90.92 (10)	O13—Ce3—Si4	29.31 (9)
O10—Ce1—Si4^i^	108.63 (9)	O14—Ce3—Si4	29.70 (8)
O14^i^—Ce1—Si4^i^	29.22 (9)	O4^iii^—Ce3—Si4	130.95 (8)
O9^ii^—Ce1—Si4^i^	86.55 (9)	O1^vii^—Ce3—Si1^iii^	96.03 (9)
O11^i^—Ce1—Si4^i^	30.83 (8)	O7—Ce3—Si1^iii^	110.56 (9)
O6—Ce1—Si2	27.50 (9)	O2^iii^—Ce3—Si1^iii^	28.21 (9)
O13—Ce1—Si2	98.21 (9)	O3^iv^—Ce3—Si1^iii^	171.84 (9)
O12—Ce1—Si2	174.13 (9)	O6—Ce3—Si1^iii^	88.46 (9)
O10—Ce1—Si2	114.33 (9)	O13—Ce3—Si1^iii^	86.88 (9)
O14^i^—Ce1—Si2	64.12 (9)	O14—Ce3—Si1^iii^	121.00 (8)
O9^ii^—Ce1—Si2	92.34 (9)	O4^iii^—Ce3—Si1^iii^	29.98 (8)
O11^i^—Ce1—Si2	112.92 (9)	Si4—Ce3—Si1^iii^	109.25 (3)
Si4^i^—Ce1—Si2	88.90 (4)	O1^vii^—Ce3—Si2^ix^	93.12 (9)
O6—Ce1—Si3	116.03 (9)	O7—Ce3—Si2^ix^	27.57 (9)
O13—Ce1—Si3	91.87 (10)	O2^iii^—Ce3—Si2^ix^	110.34 (9)
O12—Ce1—Si3	91.19 (9)	O3^iv^—Ce3—Si2^ix^	95.45 (9)
O10—Ce1—Si3	25.65 (9)	O6—Ce3—Si2^ix^	177.57 (8)
O14^i^—Ce1—Si3	109.06 (9)	O13—Ce3—Si2^ix^	108.41 (9)
O9^ii^—Ce1—Si3	172.95 (9)	O14—Ce3—Si2^ix^	62.18 (8)
O11^i^—Ce1—Si3	72.54 (8)	O4^iii^—Ce3—Si2^ix^	74.63 (9)
Si4^i^—Ce1—Si3	92.94 (3)	Si4—Ce3—Si2^ix^	88.27 (4)
Si2—Ce1—Si3	94.68 (3)	Si1^iii^—Ce3—Si2^ix^	92.53 (3)
O6—Ce1—Ce3	39.25 (9)	O1^vii^—Ce3—Si1^x^	23.77 (9)
O13—Ce1—Ce3	41.41 (9)	O7—Ce3—Si1^x^	92.53 (9)
O12—Ce1—Ce3	119.59 (9)	O2^iii^—Ce3—Si1^x^	93.20 (9)
O10—Ce1—Ce3	100.84 (9)	O3^iv^—Ce3—Si1^x^	96.79 (9)
O14^i^—Ce1—Ce3	114.11 (9)	O6—Ce3—Si1^x^	68.02 (9)
O9^ii^—Ce1—Ce3	78.88 (9)	O13—Ce3—Si1^x^	139.39 (9)
O11^i^—Ce1—Ce3	171.37 (9)	O14—Ce3—Si1^x^	157.57 (8)
Si4^i^—Ce1—Ce3	143.27 (3)	O4^iii^—Ce3—Si1^x^	64.36 (8)
Si2—Ce1—Ce3	58.60 (3)	Si4—Ce3—Si1^x^	160.04 (4)
Si3—Ce1—Ce3	105.38 (2)	Si1^iii^—Ce3—Si1^x^	78.82 (4)
O6—Ce1—Ce4^ii^	44.83 (9)	Si2^ix^—Ce3—Si1^x^	110.00 (3)
O13—Ce1—Ce4^ii^	111.23 (9)	O1^iii^—Si1—O2	112.2 (2)
O12—Ce1—Ce4^ii^	120.79 (9)	O1^iii^—Si1—O3	116.1 (2)
O10—Ce1—Ce4^ii^	167.15 (9)	O2—Si1—O3	106.2 (2)
O14^i^—Ce1—Ce4^ii^	42.09 (9)	O1^iii^—Si1—O4	108.8 (2)
O9^ii^—Ce1—Ce4^ii^	43.77 (9)	O2—Si1—O4	100.67 (19)
O11^i^—Ce1—Ce4^ii^	98.80 (8)	O3—Si1—O4	111.8 (2)
Si4^i^—Ce1—Ce4^ii^	68.76 (2)	O1^iii^—Si1—Ce2	126.94 (14)
Si2—Ce1—Ce4^ii^	53.82 (2)	O2—Si1—Ce2	45.33 (13)
Si3—Ce1—Ce4^ii^	142.11 (3)	O3—Si1—Ce2	61.23 (15)
Ce3—Ce1—Ce4^ii^	77.595 (9)	O4—Si1—Ce2	121.26 (15)
O8—Ce2—O2	107.78 (13)	O1^iii^—Si1—Ce3^iii^	125.40 (15)
O8—Ce2—O12	79.73 (13)	O2—Si1—Ce3^iii^	46.43 (13)
O2—Ce2—O12	144.59 (13)	O3—Si1—Ce3^iii^	118.24 (15)
O8—Ce2—O10	83.10 (13)	O4—Si1—Ce3^iii^	54.31 (13)
O2—Ce2—O10	142.07 (12)	Ce2—Si1—Ce3^iii^	77.32 (3)
O12—Ce2—O10	72.15 (13)	O1^iii^—Si1—Ce4^iii^	38.46 (14)
O8—Ce2—O10^iii^	152.66 (13)	O2—Si1—Ce4^iii^	79.83 (15)
O2—Ce2—O10^iii^	85.76 (13)	O3—Si1—Ce4^iii^	108.61 (15)
O12—Ce2—O10^iii^	103.20 (12)	O4—Si1—Ce4^iii^	137.53 (15)
O10—Ce2—O10^iii^	72.34 (14)	Ce2—Si1—Ce4^iii^	89.45 (3)
O8—Ce2—O13^iii^	95.55 (13)	Ce3^iii^—Si1—Ce4^iii^	114.92 (4)
O2—Ce2—O13^iii^	62.31 (12)	O1^iii^—Si1—Ce3^xi^	37.34 (13)
O12—Ce2—O13^iii^	152.92 (13)	O2—Si1—Ce3^xi^	118.77 (15)
O10—Ce2—O13^iii^	80.83 (12)	O3—Si1—Ce3^xi^	133.73 (16)
O10^iii^—Ce2—O13^iii^	69.29 (12)	O4—Si1—Ce3^xi^	71.50 (14)
O8—Ce2—O9^iii^	144.80 (13)	Ce2—Si1—Ce3^xi^	158.39 (5)
O2—Ce2—O9^iii^	77.89 (13)	Ce3^iii^—Si1—Ce3^xi^	101.18 (4)
O12—Ce2—O9^iii^	77.40 (13)	Ce4^iii^—Si1—Ce3^xi^	71.44 (3)
O10—Ce2—O9^iii^	114.27 (12)	O5—Si2—O7^i^	110.3 (2)
O10^iii^—Ce2—O9^iii^	60.08 (12)	O5—Si2—O6	113.3 (2)
O13^iii^—Ce2—O9^iii^	116.64 (12)	O7^i^—Si2—O6	105.8 (2)
O8—Ce2—O3	76.47 (13)	O5—Si2—O4^x^	113.3 (2)
O2—Ce2—O3	59.52 (11)	O7^i^—Si2—O4^x^	108.5 (2)
O12—Ce2—O3	90.43 (12)	O6—Si2—O4^x^	105.2 (2)
O10—Ce2—O3	155.27 (12)	O5—Si2—Ce1	72.52 (17)
O10^iii^—Ce2—O3	130.15 (12)	O7^i^—Si2—Ce1	111.94 (15)
O13^iii^—Ce2—O3	114.59 (11)	O6—Si2—Ce1	42.13 (13)
O9^iii^—Ce2—O3	77.30 (11)	O4^x^—Si2—Ce1	133.61 (15)
O8—Ce2—Si1	94.61 (10)	O5—Si2—Ce4^ii^	123.40 (16)
O2—Ce2—Si1	29.17 (9)	O7^i^—Si2—Ce4^ii^	48.86 (14)
O12—Ce2—Si1	118.01 (10)	O6—Si2—Ce4^ii^	57.20 (13)
O10—Ce2—Si1	169.12 (8)	O4^x^—Si2—Ce4^ii^	123.07 (14)
O10^iii^—Ce2—Si1	107.23 (9)	Ce1—Si2—Ce4^ii^	72.48 (3)
O13^iii^—Ce2—Si1	88.84 (9)	O5—Si2—Ce3^i^	67.40 (16)
O9^iii^—Ce2—Si1	73.43 (9)	O7^i^—Si2—Ce3^i^	44.01 (14)
O3—Ce2—Si1	30.51 (8)	O6—Si2—Ce3^i^	114.70 (14)
O8—Ce2—Si3^iii^	169.04 (9)	O4^x^—Si2—Ce3^i^	135.93 (15)
O2—Ce2—Si3^iii^	81.72 (10)	Ce1—Si2—Ce3^i^	89.90 (3)
O12—Ce2—Si3^iii^	89.35 (10)	Ce4^ii^—Si2—Ce3^i^	69.54 (3)
O10—Ce2—Si3^iii^	92.69 (9)	O8^v^—Si3—O9	109.9 (2)
O10^iii^—Ce2—Si3^iii^	29.96 (9)	O8^v^—Si3—O10	111.7 (2)
O13^iii^—Ce2—Si3^iii^	93.75 (9)	O9—Si3—O10	105.6 (2)
O9^iii^—Ce2—Si3^iii^	30.16 (9)	O8^v^—Si3—O11^iii^	112.5 (2)
O3—Ce2—Si3^iii^	104.81 (8)	O9—Si3—O11^iii^	110.1 (2)
Si1—Ce2—Si3^iii^	91.36 (4)	O10—Si3—O11^iii^	106.8 (2)
O8—Ce2—Si4^iv^	72.98 (10)	O8^v^—Si3—Ce2^iii^	128.57 (15)
O2—Ce2—Si4^iv^	123.96 (9)	O9—Si3—Ce2^iii^	55.43 (14)
O12—Ce2—Si4^iv^	23.56 (9)	O10—Si3—Ce2^iii^	50.22 (14)
O10—Ce2—Si4^iv^	93.93 (9)	O11^iii^—Si3—Ce2^iii^	118.79 (15)
O10^iii^—Ce2—Si4^iv^	119.59 (8)	O8^v^—Si3—Ce4	53.26 (15)
O13^iii^—Ce2—Si4^iv^	167.97 (9)	O9—Si3—Ce4	56.77 (15)
O9^iii^—Ce2—Si4^iv^	75.37 (9)	O10—Si3—Ce4	121.00 (14)
O3—Ce2—Si4^iv^	66.98 (8)	O11^iii^—Si3—Ce4	132.16 (15)
Si1—Ce2—Si4^iv^	95.56 (3)	Ce2^iii^—Si3—Ce4	92.12 (4)
Si3^iii^—Ce2—Si4^iv^	97.33 (3)	O8^v^—Si3—Ce1	71.73 (15)
O8—Ce2—Ce1^iii^	132.77 (9)	O9—Si3—Ce1	112.56 (14)
O2—Ce2—Ce1^iii^	56.85 (9)	O10—Si3—Ce1	40.86 (13)
O12—Ce2—Ce1^iii^	140.76 (9)	O11^iii^—Si3—Ce1	132.06 (15)
O10—Ce2—Ce1^iii^	88.69 (9)	Ce2^iii^—Si3—Ce1	71.85 (3)
O10^iii^—Ce2—Ce1^iii^	37.59 (8)	Ce4—Si3—Ce1	90.17 (3)
O13^iii^—Ce2—Ce1^iii^	37.25 (9)	O12^iv^—Si4—O13	113.3 (2)
O9^iii^—Ce2—Ce1^iii^	80.15 (8)	O12^iv^—Si4—O14	117.4 (2)
O3—Ce2—Ce1^iii^	115.42 (8)	O13—Si4—O14	105.4 (2)
Si1—Ce2—Ce1^iii^	85.07 (3)	O12^iv^—Si4—O11	108.3 (2)
Si3^iii^—Ce2—Ce1^iii^	56.89 (2)	O13—Si4—O11	109.4 (2)
Si4^iv^—Ce2—Ce1^iii^	154.20 (3)	O14—Si4—O11	102.3 (2)
O5—Ce4—O1	85.88 (13)	O12^iv^—Si4—Ce1^ix^	124.17 (16)
O5—Ce4—O3^v^	89.90 (13)	O13—Si4—Ce1^ix^	122.54 (15)
O1—Ce4—O3^v^	147.59 (13)	O14—Si4—Ce1^ix^	48.06 (14)
O5—Ce4—O7^vi^	142.34 (14)	O11—Si4—Ce1^ix^	54.50 (13)
O1—Ce4—O7^vi^	89.55 (12)	O12^iv^—Si4—Ce3	122.57 (15)
O3^v^—Ce4—O7^vi^	74.52 (12)	O13—Si4—Ce3	52.68 (14)
O5—Ce4—O7^vii^	79.14 (14)	O14—Si4—Ce3	54.71 (15)
O1—Ce4—O7^vii^	68.22 (12)	O11—Si4—Ce3	129.08 (15)
O3^v^—Ce4—O7^vii^	79.42 (12)	Ce1^ix^—Si4—Ce3	92.60 (4)
O7^vi^—Ce4—O7^vii^	64.55 (13)	O12^iv^—Si4—Ce2^iv^	36.72 (14)
O5—Ce4—O8^v^	66.01 (13)	O13—Si4—Ce2^iv^	106.37 (15)
O1—Ce4—O8^v^	128.92 (13)	O14—Si4—Ce2^iv^	86.82 (14)
O3^v^—Ce4—O8^v^	77.17 (12)	O11—Si4—Ce2^iv^	138.90 (15)
O7^vi^—Ce4—O8^v^	138.50 (12)	Ce1^ix^—Si4—Ce2^iv^	118.44 (4)
O7^vii^—Ce4—O8^v^	137.57 (12)	Ce3—Si4—Ce2^iv^	88.97 (3)
O5—Ce4—O14^vi^	134.07 (13)	Si1^iii^—O1—Ce3^vii^	118.88 (19)
O1—Ce4—O14^vi^	135.66 (12)	Si1^iii^—O1—Ce4	117.4 (2)
O3^v^—Ce4—O14^vi^	65.09 (12)	Ce3^vii^—O1—Ce4	113.89 (15)
O7^vi^—Ce4—O14^vi^	69.81 (12)	Si1—O2—Ce2	105.51 (18)
O7^vii^—Ce4—O14^vi^	127.90 (12)	Si1—O2—Ce3^iii^	105.37 (18)
O8^v^—Ce4—O14^vi^	70.98 (12)	Ce2—O2—Ce3^iii^	114.04 (15)
O5—Ce4—O9	101.90 (14)	Si1—O3—Ce4^v^	122.03 (19)
O1—Ce4—O9	89.74 (12)	Si1—O3—Ce3^iv^	125.0 (2)
O3^v^—Ce4—O9	122.51 (12)	Ce4^v^—O3—Ce3^iv^	98.17 (13)
O7^vi^—Ce4—O9	115.46 (11)	Si1—O3—Ce2	88.26 (16)
O7^vii^—Ce4—O9	157.89 (12)	Ce4^v^—O3—Ce2	98.75 (13)
O8^v^—Ce4—O9	58.52 (12)	Ce3^iv^—O3—Ce2	123.54 (14)
O14^vi^—Ce4—O9	66.93 (12)	Si2^xi^—O4—Si1	129.2 (2)
O5—Ce4—O6^viii^	150.12 (13)	Si2^xi^—O4—Ce3^iii^	133.16 (19)
O1—Ce4—O6^viii^	70.33 (11)	Si1—O4—Ce3^iii^	95.71 (16)
O3^v^—Ce4—O6^viii^	119.89 (11)	Si2—O5—Ce4	138.9 (2)
O7^vi^—Ce4—O6^viii^	57.87 (11)	Si2—O6—Ce1	110.37 (18)
O7^vii^—Ce4—O6^viii^	106.96 (11)	Si2—O6—Ce3	115.34 (19)
O8^v^—Ce4—O6^viii^	115.35 (11)	Ce1—O6—Ce3	104.53 (14)
O14^vi^—Ce4—O6^viii^	65.44 (11)	Si2—O6—Ce4^ii^	93.40 (16)
O9—Ce4—O6^viii^	61.50 (11)	Ce1—O6—Ce4^ii^	98.43 (12)
O5—Ce4—Si3	82.23 (11)	Ce3—O6—Ce4^ii^	132.92 (14)
O1—Ce4—Si3	110.48 (9)	Si2^ix^—O7—Ce3	108.42 (18)
O3^v^—Ce4—Si3	100.71 (9)	Si2^ix^—O7—Ce4^xii^	102.57 (19)
O7^vi^—Ce4—Si3	133.72 (8)	Ce3—O7—Ce4^xii^	98.63 (13)
O7^vii^—Ce4—Si3	161.37 (9)	Si2^ix^—O7—Ce4^vii^	123.2 (2)
O8^v^—Ce4—Si3	28.79 (9)	Ce3—O7—Ce4^vii^	105.80 (14)
O14^vi^—Ce4—Si3	66.87 (9)	Ce4^xii^—O7—Ce4^vii^	115.45 (13)
O9—Ce4—Si3	29.77 (8)	Si3^v^—O8—Ce2	138.7 (2)
O6^viii^—Ce4—Si3	89.27 (8)	Si3^v^—O8—Ce4^v^	97.95 (18)
O5—Ce4—Si2^viii^	163.01 (10)	Ce2—O8—Ce4^v^	107.50 (15)
O1—Ce4—Si2^viii^	80.50 (9)	Si3—O9—Ce1^viii^	117.11 (19)
O3^v^—Ce4—Si2^viii^	96.17 (9)	Si3—O9—Ce2^iii^	94.41 (17)
O7^vi^—Ce4—Si2^viii^	28.57 (9)	Ce1^viii^—O9—Ce2^iii^	130.18 (16)
O7^vii^—Ce4—Si2^viii^	86.37 (9)	Si3—O9—Ce4	93.46 (17)
O8^v^—Ce4—Si2^viii^	130.82 (8)	Ce1^viii^—O9—Ce4	96.94 (13)
O14^vi^—Ce4—Si2^viii^	62.47 (8)	Ce2^iii^—O9—Ce4	119.87 (14)
O9—Ce4—Si2^viii^	88.21 (8)	Si3—O10—Ce1	113.49 (19)
O6^viii^—Ce4—Si2^viii^	29.40 (8)	Si3—O10—Ce2	124.03 (18)
Si3—Ce4—Si2^viii^	112.00 (3)	Ce1—O10—Ce2	105.83 (14)
O5—Ce4—Si1^iii^	79.59 (10)	Si3—O10—Ce2^iii^	99.82 (17)
O1—Ce4—Si1^iii^	24.16 (9)	Ce1—O10—Ce2^iii^	104.00 (12)
O3^v^—Ce4—Si1^iii^	166.44 (8)	Ce2—O10—Ce2^iii^	107.66 (14)
O7^vi^—Ce4—Si1^iii^	108.85 (9)	Si3^iii^—O11—Si4	128.8 (2)
O7^vii^—Ce4—Si1^iii^	90.11 (9)	Si3^iii^—O11—Ce1^ix^	136.4 (2)
O8^v^—Ce4—Si1^iii^	105.82 (9)	Si4—O11—Ce1^ix^	94.67 (15)
O14^vi^—Ce4—Si1^iii^	128.47 (9)	Si4^iv^—O12—Ce2	119.7 (2)
O9—Ce4—Si1^iii^	68.63 (8)	Si4^iv^—O12—Ce1	124.8 (2)
O6^viii^—Ce4—Si1^iii^	71.29 (8)	Ce2—O12—Ce1	110.77 (15)
Si3—Ce4—Si1^iii^	86.45 (3)	Si4—O13—Ce1	131.2 (2)
Si2^viii^—Ce4—Si1^iii^	91.70 (3)	Si4—O13—Ce3	98.00 (18)
O1^vii^—Ce3—O7	71.08 (13)	Ce1—O13—Ce3	100.78 (13)
O1^vii^—Ce3—O2^iii^	115.42 (13)	Si4—O13—Ce2^iii^	118.1 (2)
O7—Ce3—O2^iii^	134.51 (12)	Ce1—O13—Ce2^iii^	101.75 (14)
O1^vii^—Ce3—O3^iv^	81.85 (13)	Ce3—O13—Ce2^iii^	101.24 (13)
O7—Ce3—O3^iv^	76.31 (12)	Si4—O14—Ce1^ix^	102.72 (18)
O2^iii^—Ce3—O3^iv^	147.15 (12)	Si4—O14—Ce4^xii^	143.4 (2)
O1^vii^—Ce3—O6	84.56 (12)	Ce1^ix^—O14—Ce4^xii^	99.49 (13)
O7—Ce3—O6	150.12 (11)	Si4—O14—Ce3	95.60 (18)
O2^iii^—Ce3—O6	71.43 (12)	Ce1^ix^—O14—Ce3	131.95 (15)
O3^iv^—Ce3—O6	83.50 (12)	Ce4^xii^—O14—Ce3	90.65 (12)

Symmetry codes: (i) *x*−1, *y*, *z*; (ii) *x*, *y*−1, *z*; (iii) −*x*+1, −*y*+1, −*z*+1; (iv) −*x*+1, −*y*, −*z*+1; (v) −*x*, −*y*+1, −*z*+1; (vi) *x*−1, *y*+1, *z*; (vii) −*x*+1, −*y*+1, −*z*; (viii) *x*, *y*+1, *z*; (ix) *x*+1, *y*, *z*; (x) *x*, *y*, *z*−1; (xi) *x*, *y*, *z*+1; (xii) *x*+1, *y*−1, *z*.
